# Call for Papers: PLOS Medicine Special Issue on Bacterial Antimicrobial Resistance—Surveillance and Prevention

**DOI:** 10.1371/journal.pmed.1004014

**Published:** 2022-05-17

**Authors:** The PLOS Medicine Editors

**Affiliations:** Public Library of Science, San Francisco, California, United States of America

The editors of *PLOS Medicine*, together with guest editors Timothy Walsh, Ramanan Laxminarayan and Ana Cristina Gales, announce a forthcoming Special Issue dedicated to bacterial antimicrobial resistance (AMR). Research submissions are now being invited.

The emergence of pathogenic bacteria which cannot be effectively treated with existing drugs has been prioritised by the World Health Organization as one of the top ten global public health threats facing humanity [1]. Drug-resistant infections are associated with substantial morbidity and mortality, and were estimated to contribute to 4.95 million deaths globally in 2019 [2]. The burden of antimicrobial resistance (AMR) is disproportionately observed in low- and middleincome countries (LMICs), particularly sub-Saharan Africa [2]. Without intervention, it has been estimated that global deaths attributable to AMR could reach 10 million annually by 2050 [3].

AMR is a One Health problem and its causes lie in human, animal and environmental domains. The overuse and misuse of antibiotics, and the potential for transmission within and between these domains is responsible for the rapid global spread of drug-resistant pathogens. Use of antibiotics increased by 65% globally between 2000 and 2015, and more than doubled in LMICs over the same period [4]. Pathogen AMR evolution can limit the effectiveness of available antibiotics and far outpaces our ability to develop new drugs. Of the 32 antibiotics in clinical development to tackle priority pathogens in 2019, only six were classified as innovative [5]. Action to impede the development of drug-resistance is urgently required.

The guest editors and *PLOS Medicine* editors seek high-quality and high-impact research submissions related to the main drivers, surveillance and prevention of bacterial antimicrobial resistance, particularly in low- and middle-income settings. Areas of particular interest include the prevalence and clinical challenges of drug-resistant bacteria, interventions to reduce disease transmission, diagnostics informing antimicrobial prescribing, misuse and overuse of antimicrobials, economics of antimicrobial access and use, and One Health interventions to reduce AMR. Submission of articles related to pathogens of highest concern and highest global burden (excluding *Mycobacterium tuberculosis*) are strongly encouraged.

Please see plos.io/AMR for more detailed information.

To submit your manuscript for consideration, please visit http://journals.plos.org/plosmedicine/s/submit-now, indicating your interest in the Special Issue in your cover letter. Questions about the Special Issue can be directed to plosmedicine@plos.org.

The submission deadline is July 15^th^ 2022.
